# Middlesex Hospital

**Published:** 1829-11

**Authors:** 


### HOSPITAL REPORTS.

### MIDDLESEX HOSPITAL.

### *Case of Lithotomy.*

RICHARD MARSHALL, ætat. four years, was admitted into the Middlesex Hospital, under the care of Mr. MAYO, on the 12th of September. His parents stated that he had laboured under his present symptoms, which consist of frequent and painful micturition, for a year. On sounding him, a stone was felt. At the time of his admission, the bowels were much disordered; he had frequent stools, some wholly blood and mucus, and with each the rectum was inverted for several inches.—℞. Confect. Aromat. gr. viij. cum Pulv. Cretæ cum Opio gr. v. ex Aquâ Cinnamomi, sextis horis.

In two days the action of the bowels became regular, and the patient subsequently took, as his only medicine, equal parts of milk and lime-water twice a day.

The lateral operation was performed by Mr. Mayo on the 17th, and two smooth, flat, oval stones were extracted; the largest about an inch and a half in its long diameter. After the operation, (as in another case, in which we saw Mr. Mayo operate a few months since,) no dressing was applied to the wound, beside a piece of lint dipped in oil.

An hour after the operation, no water having passed through the wound, Mr. Mayo drew the lips of the wound apart, and passed his finger to the bladder. In the evening, however, the child made water freely through the natural passage, no water passing out at the wound. The child took at night two grains of James's powder, but was restless.

Early the next morning, no water having yet flowed through the wound, the wound was again opened with the finger. The child then strove to make water, when a little passed through the urethra, the greater part with a gush through the wound.

The water continued to flow through the wound the two following days.

On the 21st, the water passed again by the natural passage alone. The wound now healed rapidly, and the child was discharged well on the 13th of October.

Mr. Mayo observes, that, where death occurs after lithotomy, the most frequent cause is effusion of urine about the neck of the bladder; and that urine may lodge in the pelvis, either through the internal incisions and general separation of parts being too free, or in consequence of the wound uniting superficially by adhesion. The latter risk may be avoided, either by opening the wound with the finger, if the urine does not pass through it soon after the operation; or by introducing a slip of lint dipped in oil into the wound, as soon as the operation is over; or by leaving a piece of gum catheter, passed through the wound, with its extremity in the bladder. Mr. Mayo prefers the plan of gently opening the wound once, or oftener if necessary, during the twenty-four hours following the operation; upon the supposition that the wound heals more readily when this method is adopted, than when either of the others is followed.

---

### *Menstruation during Pregnancy.*

Sarah Cooper, ætat. thirty, is a patient in the Middlesex Hospital, with varicose veins of the leg, which have been partially obliterated by the use of the caustic paste. Mr. Mayo pointed out the following circumstances in her history, as worthy of being put on record:

She was married at the age of seventeen, shortly after the catamenia had commenced. She has borne nine children, and had two miscarriages. When not pregnant, the catamenia recur regularly at the usual period, preceded, for twenty-four hours, by pain at the epigastrium and in the back, and a sense of shooting in the breasts. The entire indisposition lasts a week. When she is suckling, the catamenia occur exactly in the same manner as when she is neither suckling nor pregnant. During the whole term of her pregnancy, the catamenia recur more frequently, more profusely, and less regularly; about every third week. She has become pregnant five times while suckling.

